# P-1678. Gram-Negative Rod Bacteremia due to Organisms not Detected on the BioFire® Blood Culture Identification 2 (BCID2) Panel: Time to Effective Antimicrobial Therapy

**DOI:** 10.1093/ofid/ofaf695.1852

**Published:** 2026-01-11

**Authors:** Bryan O Navarro, Gerard Gawrys, Amber Welborn, Christopher R Frei

**Affiliations:** The Ohio State University Wexner Medical Center, Grandview Heights, OH; University Health, San Antonio, Texas; University Health, San Antonio, Texas; University of Texas, San Antonio, Texas

## Abstract

**Background:**

The BioFire® BCID2 panel has revolutionized rapid diagnostics, reducing time to organism identification and targeted therapy. Despite detectability of 16 additional organisms and resistance genes compared to the original panel, limited data exists regarding the clinical impact of BCID-2 negative Gram-Negative Rod (GNR) bacteremia cases.

**Methods:**

We evaluated the effect of BCID2-negative GNR bacteremia on time to effective therapy (TTET). Overall, 135 patients had BCID2-negative GNR bacteremia from July 2020 to August 2024. Further analysis examined the distribution of BCID-2 negative GNR pathogens and compared clinical outcomes between aerobic and anaerobic cases. Descriptive statistics were used to summarize patient characteristics. Chi-square and Wilcoxon Rank Sum tests were used to compare health outcomes. P-values were statistically significant if they were less than a pre-specified alpha level of 0.05.

**Results:**

At 12 hours post-BCID2 result, 24% (32/135) of patients lacked effective therapy. This group consisted of 15 aerobes and 17 anaerobes, with a median (interquartile range) TTET of 41 (20-51) hours. A 20-hour increase in median TTET was observed in the aerobic group vs. the anaerobic group: 47 (39-59) vs. 27 (18-42), p=0.02. Aerobes not on effective therapy were led by *Pseudomonas putida* (13%), *Achromobacter spp.* (13%), *Brucella anthropi* (13%), and *Myroides spp.* (13%). Despite these findings, a 7-day increase in median hospital length of stay (LOS) was observed among anaerobes: (17 (10-34) vs. 10 (7-19) days, p=0.01), with *Bacteroides thetaiotaomicron* (n=23) and *Fusobacterium spp.* (n=27) accounting for 60% (50/83) of the anaerobic pathogens.

**Conclusion:**

These findings indicate that nearly 1 in 4 patients lacked effective therapy 12 hours after a negative BCID2 result. Over half of these cases involved anaerobic pathogens, particularly *Bacteroides thetaiotaomicron* and *Fusobacterium spp.*, which were associated with a prolonged hospital LOS.

**Disclosures:**

Christopher R. Frei, PharmD, FCCP, BCPS, Amgen: Grant/Research Support|AstraZeneca: Grant/Research Support

